# Leaving Home and Destination of Early Nest Leavers: Ethnicity, Spaces and Prices

**DOI:** 10.1007/s10680-016-9375-3

**Published:** 2016-02-17

**Authors:** Aslan Zorlu, Ruben van Gaalen

**Affiliations:** 1 Department of Human Geography, Amsterdam Institute for Social Science Research, University of Amsterdam, Nieuwe Achtergracht 166, 1018 WV Amsterdam, The Netherlands; 2 Statistics Netherlands, Henri Faasdreef 312, 2492 JP Den Haag, The Netherlands; 3 Department of Sociology, University of Amsterdam, Nieuwe Achtergracht 166, 1018 WV Amsterdam, The Netherlands

**Keywords:** Migrants, Transition to adulthood, Housing quality, Location choice

## Abstract

This study examines ethnic differences in leaving the parental home and the choice of destination (both location and quality of housing) in the Netherlands. Using unique individual administrative panel data, we study the mobility of the entire birth cohort 1983. In contrast to previous studies, this paper includes the geographical location and the quality of destination living arrangements in the analysis, in an attempt to explain ethnic differences in leaving the parental home. We show that ethnic minority youth, in particular those from Turkish and Moroccan origin, improve their housing quality when leaving the parental home. This results in earlier home-leaving than among young people of native Dutch origin. Our results on the early home-leaving behaviour of ethnic minority youth are robust with regard to the geographical distance of nest leavers.

## Introduction

A substantial number of studies have considered the process of leaving the parental home as an outcome of demographic transitions, social pressures, economic factors, institutional structures and social norms (Goldscheider et al. 1993; Nilsson and Strandh 1999; Ermisch 1999; Mulder and Hooimeijer 2002; Aassve et al. 2002; Bernhardt et al. 2005). However, the literature on home-leaving behaviour among ethnic minority groups in western countries is less developed. The existing studies reveal mixed results about ethnic differences in co-residence rates with parents. Whereas studies relying on survey data suggest a higher probability of co-residence with parents for ethnic minority youth from a disadvantaged background (De Valk and Billari 2007; de Valk and Liefbroer 2007a; Bolt 2002), evidence based on Dutch register data indicates the opposite. Zorlu and Mulder (2011) show that young people from the major ethnic minority groups in the Netherlands (Turkish, Moroccan and Surinamese) leave the parental home at younger ages than young native Dutch people. Most studies have used explanatory variables related to socio-demographic conditions in the parental home. The living arrangements after leaving home have never been fully addressed. A rare exception is the work of Zorlu and Mulder (2010), who examined the potential implications of leaving home for ethnic residential diffusion. They report that nest leavers tend to choose neighbourhoods with a similar ethnic composition to the one where their parental home is located.

Most studies focus on the relationship between parental home characteristics and nest leaving (e.g. Mulder and Hooimeijer 2002; Nilsson and Strandh 1999; Ermisch 1999; Whittington and Peters 1996). The literature has extensively explored the effects of conditions in the parental home, but the characteristics of destination locations have largely been ignored (Zorlu and Mulder 2011; Leopold et al. 2012). This is perhaps due to a lack of information about destination housing. Comparing origin and destination locations and housing qualities will improve our understanding of the decision-making process of nest leavers from various ethnic origins. Most leaving home literature suggests that nest leavers face a decline in housing quality while gaining more autonomy (Mulder and Hooimeijer 2002). A parental home that offers better housing quality seems to keep many potential nest leavers at home (Mulder 2013). This trade-off is potentially different for nest leavers from ethnic minority groups whose parental home quality may be significantly lower than that of Dutch nest leavers (Zorlu et al. 2014). For example, ethnic minority families may share relatively few economic resources with more individuals in smaller houses. So nest leavers from ethnic minority families may on average give up less housing quality to gain more autonomy.

This study contributes to earlier work by examining ethnic differences regarding the leaving home process in relation to the location and qualitative characteristics of the parental home and the home of destination. Our analysis is based on individual administrative panel data from the Netherlands, covering the period from 1999 until 2005. A complete birth cohort of 16-year olds in 1999 from various ethnic backgrounds is followed in time to assess choices of nest leavers regarding timing and destinations.

## Migrants in the Netherlands

Migrants in the Netherlands can be grouped into six categories of origin countries regarding their population size: Turks, Moroccans, Surinamese, Antilleans, other non-western and western migrants. Turkish and Moroccans came to the Netherlands as ‘guest workers’ in the 1960s. Immigration flows from Suriname and the Dutch Antilles are the result of colonial relations. Immigration from developed western countries has been related to economic conditions and fluctuates with the economic situation (De Beer 2003). The category of *other non*-*western* covers a variety of remaining immigrants from developing countries who do not belong to the main groups.

This historical background reflects the socio-economic position of these groups and their cultural distance to the host society. Surinamese and Antillean (*Caribbean*) migrants tend to speak Dutch and have cultural norms that are similar to the mainstream society due to the colonial relations between the countries. The predominantly Muslim community of Turkish and Moroccan (*Mediterranean*) migrants is relatively less educated, did not speak the language prior to immigration and their cultural distance from the majority population tends to be greater. The category *other non*-*western* is a highly heterogeneous group which may be more similar to Turkish and Moroccan migrants regarding their characteristics measured in this study such as education, income, parental characteristics and neighbourhood characteristics (see Table 1b). *Western* migrants are often quite comparable with native Dutch people (Zorlu 2013).

**Table 1:** Variables and their route-specific means at the time of exit or censoring

		Mean	SD	Min	Max
*Pathways*					
Union	Leaving home for union	0.17	0.38	0	1
Independent	Leaving home for independent	0.26	0.44	0	1
Shared	Leaving home for shared	0.05	0.21	0	1
Stayed home	Not left home yet	0.53	0.50	0	1
*Age and gender*					
*t* + 1	Age 17	0.08	0.27	0	1
*t* + 2	Age 18	0.10	0.30	0	1
*t* + 3	Age 19	0.11	0.31	0	1
*t* + 4	Age 20	0.11	0.31	0	1
*t* + 5	Age 21	0.58	0.49	0	1
Girl	1 if girl	0.49	0.50	0	1
*Immigrant origin*	The reference group = native				
Moroccan	1 if originated from Morocco	0.03	0.17	0	1
Turkish	1 if originated from Turkey	0.03	0.17	0	1
Surinamese	1 if originated from Suriname	0.03	0.17	0	1
Antillean	1 if originated from the Dutch Antilles/Aruba	0.01	0.10	0	1
Other non-western	1 if originated from other non-western countries	0.03	0.18	0	1
Western	1 if originated from other western countries	0.07	0.26	0	1
Second generation	1 if born in NL and both parents were born abroad	0.08	0.27	0	1
Second gen. (mixed)	1 if born in NL and one parent was born abroad	0.06	0.25	0	1
Individual char					
In education	1 if in education	0.51	0.50	0	1
Graduated in HE	1 if graduated in higher education	0.02	0.14	0	1
Employed	1 if employed	0.48	0.50	0	1
Monthly income	Monthly income in 100 euros	7.91	6.43	0	89
Owner-occupied	1 if owner-occupied parental home	0.64	0.48	0	1
Value of dwelling	Value of dwelling for individual in 1000 euros	38.91	28.33	0	2,799
Family structure					
No. of adults in HH	Number of adults in household	1.81	0.51	0	10
No. of children in HH	Number of kids in household	2.00	1.22	0	16
Mother married	The reference group				
Mother unmarried	1 if mother unmarried	0.03	0.17	0	1
Mother widow	1 if mother widow	0.02	0.15	0	1
Mother divorced	1 if mother divorced	0.13	0.34	0	1
Parents econ.	The reference group = non-participant				
Mother employed	1 if mother employed	0.55	0.50	0	1
Mother self-employed	1 if mother self-employed	0.06	0.24	0	1
Mother welfare	1 if mother has welfare benefit	0.10	0.30	0	1
Mother benefit	1 if mother has another benefit	0.04	0.20	0	1
Father employed	1 if father employed	0.67	0.47	0	1
Father self-employed	1 if father self-employed	0.10	0.30	0	1
Father welfare	1 if father has welfare benefit	0.09	0.29	0	1
Father benefit	1 if father has another benefit	0.05	0.22	0	1
Income mother	Monthly income of mother in 100 euros	10.67	10.54	0	89
Income father	Monthly income of father in 100 euros	27.20	18.04	0	102
Local conditions					
Urbanised I	Very strongly urbanised municipality	0.14	0.35	0	1
Urbanised II	Strongly urbanised in municipality	0.26	0.44	0	1
Urbanised III	Moderately urbanised in municipality	0.22	0.41	0	1
Urbanised VI/V	Weakly urbanised (the reference group)	0.08	0.27	0	1
Non-western (%)	Non-western in the neighbourhood (%)	9.62	13.68	0	91
μ value houses	Mean value of houses in the neighbourhood	1,47,983 €	1,73,459 €	0	285 M€
Benefits (%)	Benefit recipients in the neighbourhood (%)	10.50	4.38	0	75
*N*		1,72,288			

*Source*: SSD, own calculations

These groups of immigrant origin tend to differ significantly in many respects, such as the degree of traditionalism and their social and economic position in the Netherlands (De Valk and Liefbroer 2007a; Schans 2007). These differences generate different home-leaving patterns (De Valk and Liefbroer 2007b). In the non-western countries of origin, family-related individual decisions are often prone to familial and religious concerns, while in secularised and individualised Dutch society, individuals mostly make autonomous decisions about the timing of transitions into adulthood. Although Caribbean and Mediterranean migrants are more family-oriented than the native Dutch, there are fundamental differences between Caribbean and Mediterranean migrants with regard to the timing and mechanisms of family life transitions such as leaving home, marriage and child-bearing (De Valk and Liefbroer 2007a). In Turkish and Moroccan communities, the dominant living arrangement is thought to be leaving home to get married. There are more traditionally arranged marriages, and Turkish and Moroccan youth marry and have their first child younger than Caribbean migrants. Koc (2007) showed that the home-leaving process in Turkey follows a sequential pattern like many other Mediterranean countries: entry into the labour force, marriage, the birth of the first child and finally leaving home. It is likely that cultural norms facilitate a delayed departure from the parental home and an extended family life in which an intergenerational transmission of resources takes place. In the Caribbean tradition, unmarried cohabitation, child-bearing out of wedlock and single-mother families are more common (De Valk and Liefbroer 2007b). The next section reviews the relevant literature, and two hypotheses regarding ethnic differences are formulated regarding the location and quality of the destination home of nest leavers.

## Related Literature and Hypotheses

Extensive research has been done on the determinants of leaving the parental home in western countries. Much of this research deals with the pathways out of home (such as marriage, education, and labour market participation) and their trigger roles in determining the decision to leave home (e.g. Goldscheider et al. 1993; Bernhardt et al. 2005). Other studies focus on the opportunities and constraints within the parental home and in the labour and housing markets (e.g. Mulder and Hooimeijer 2002; Nilsson and Strandh 1999; Ermisch 1999; Whittington and Peters 1996). Still other studies focused on differences in the patterns and timing of leaving home between generations, regions within countries, and across countries, according to the degree of traditionalism, individualisation and organisation of the welfare state (Giuliano 2007; Aassve et al. 2002; Goldscheider et al. 1993; Buck and Scott 1993; Aquilino 1991). Some studies have paid attention to the varying patterns of leaving home among young people from migrant families (Nilsson and Strandh 1999 for Sweden; Bolt 2002; Zorlu and Mulder 2011; De Valk and Billari 2007 for the Netherlands; Glick and Van Hook 2002 for the USA).

Ethnic differences in leaving home patterns are associated with the specific position of migrants between the cultures of the origin and the host society. Cultural norms refer to dominant cultural norms and preferences in a migrant’s country of origin in the timing and routes of departure from the parental home. In the countries of origin of non-western migrants, decisions regarding the timing of transitions into adulthood are often prone to familial and religious concerns. Reasoning along this line suggests that young people from more conservative non-western migrant communities, such as the Turkish and Moroccan, would be older when they leave home and keep living with their parents longer. Survey data on ethnic minority young people in the Netherlands confirm this expectation (Bolt 2002; De Valk and Billari 2007; De Valk and Liefbroer 2007a). However, Dutch register data indicate an opposite outcome: Non-western migrant male and female adolescents leave the parental home at younger ages than their Dutch counterparts (Zorlu and Mulder 2011). The most deviant pattern is observed for Mediterranean (Turkish and Moroccan) migrants who are grouped in the same categories as in this study.

The extensive leaving home literature emphasises gender differences in both timing and the pathways out of the parental home. Young women leave home earlier than young men and more often to form a union. This pattern also holds for ethnic minority groups. With regard to gender differences, the literature points at the important role of union formation of women, in particular women from disadvantaged ethnic minority groups (Kleinepier and de Valk 2015; Mulder 2013). Previous research also concerns the link between the nature of family relations and the proximity of residential locations of nest leavers to parents (Mulder 2013; Leopold et al. 2012). In conservative (rural) communities, daughters tend to escape to urban areas to gain more personal autonomy. On the other hand, daughters are more likely to invest more in family relations than sons. These two motivations are likely related to the pathways out of home. Young women who leave home to gain more autonomy are likely to choose accommodation in a more urban area far away. Alternatively, young women will either stay at home longer or leave home for nearby accommodation to keep contacts with the family.

In the leaving home literature, attempts have been made to use differences in timing and pathways to explain variations in characteristics of nest leavers measured when the individuals lived with their parents. The focus of previous studies has been predominantly on the role of individual and household characteristics, the housing market and the national context. Leaving home research has largely neglected the role of the destination living arrangement, although individual decisions are potentially based on both the quality of the original housing accommodation (the parental home) and the quality of destination housing. Information about potential destination living arrangement has not been often used. An exception is the work by Zorlu and Mulder (2010) who studied ethnic differences in the choice of the destination neighbourhood among nest leavers with a particular focus on ethnic residential segregation. Their study showed that ethnic minority nest leavers tend to choose neighbourhoods similar to their parents’ and found little evidence of spatial diffusion among the nest leavers.

Two factors are important when considering the destination living arrangement. Firstly, nest leavers will choose their new home somewhere far away from the parental home if they want to escape daily parental control and to gain more independence and privacy. This implies that many young people from ethnic minorities in large cities ought to move to less urbanised regions, or to another large city, if they want to escape from parental control. It is likely that they have difficulties avoiding co-ethnics and be anonymous even in large cities since ethnic social networks may be relatively strong. But such mobility is unlikely since young people generally tend to move to large cities. Young people from ethnic minorities living in less urbanised areas are more likely to leave home for a living arrangement in more urbanised locations if they want to escape parental control. With regard to the transition to adulthood, evidence suggests that geographical distance has profound implications for the intensity of family relations. Young adults who left home for an accommodation within five kilometres from their parents are more likely to receive parental support (Mulder and Van der Meer 2009; Knijn and Liefbroer 2006). Despite physical independence from parents, such a small distance enables a certain degree of parental control. On the other hand, leaving home for longer distances is associated with less support from parents and more likelihood of escaping parental control. It may also have long-term implications for family contacts and solidarity. Greater distances between young adults and their parents can lead to less support and less intergenerational contacts which may in turn have detrimental effects on the strength of affective ties (Leopold et al. 2012; Bucx et al. 2008).

Since parental controls are likely to be more intense in conservative immigrant communities, than in the secularised and individualised Dutch society (Billari and Liefbroer 2007; De Valk and Billari 2007; Kleinepier and de Valk 2015), young adults from ethnic minorities must move out to an accommodation far from their parents to evade parental authority. If they prefer to keep intense intergenerational interaction, they will move out to an accommodation close to the parental home. Our first hypothesis relates to the early home-leaving behaviour of ethnic minority youth to the location of destination housing from the parental home.

### **H1**

Young adults from ethnic minorities tend to leave home at younger ages for a destination living arrangement in another urbanisation area, further away from the parental home than their Dutch counterparts.

Secondly, nest leavers usually sacrifice some housing quality when they leave the parental home which usually has more amenities and offers greater quality (Murphy and Wang 1998). Independent nest leavers typically move to a (small) rental room or a small rental house. This type of housing is associated with low quality, while the parental home usually includes more facilities and more physical comfort or quality measured as the monetary value of house. Evidence suggests that a parental home offering a higher housing quality seems to discourage potential home leavers from moving out for an independent accommodation close to home (Mulder 2013). This does not hold for nest leavers who go to college, who seem to rely on parental affluence as an extra resource (Mulder and Clark 2000). Non-western ethnic minority groups in the Netherlands tend to live in relatively low-quality rental houses, usually in the same neighbourhoods of large cities (Zorlu 2013; Zorlu and Mulder 2008). This implies that ethnic minority nest leavers will lose less quality, or perhaps even gain more quality than the native Dutch, because their parental homes were already of a low quality. Native Dutch nest leavers will lose more quality since they enjoy more quality in the parental home and their destination home will be of a lower quality. Quality gains or limited quality losses may encourage ethnic minority youth to leave the parental home at a younger age while Dutch youth may stay at the parental home longer to avoid larger quality losses. Our second hypothesis explores differences in the relationship between the timing of leaving home and potential quality losses across ethnic groups.

### **H2**

Young adults from ethnic minorities tend to leave home younger because they will obtain more quality from leaving home than young native Dutch adults due to the relatively poor quality of their parental home.

The change in the housing quality of nest leavers is expected to follow socio-economic position of ethnic minority groups in the Netherlands. Turkish and Moroccan nest leavers will either improve their housing quality or suffer the least quality loss. Surinamese and Antillean nest leavers will suffer some quality loss. Dutch and western nest leavers are expected to face most quality loss.

## Data

For the analysis, we used the entire birth cohort of people who were 16 on the last Friday of September 1999 and followed them until 2006. Data come from the System of Social statistical Datasets (SSD) hosted by Statistic Netherlands. The SSD is an integrated, longitudinal database of numerous registers and surveys, containing the most important socio-economic and socio-demographic variables—checked for consistency—of the complete population of the Netherlands (Bakker et al. 2014). All individuals from the age cohort 16 co-reside with one of the parents in 1999. A very limited number of young individuals, <0.5 % of the cohort who did not co-reside with parent(s) (most live in institutional or foster homes), is excluded from the analysis. This ensures an accurate tracking over time of the mobility of young people at risk of leaving the parental home.

### Variables and Descriptive Statistics

Our dependent variable, the *event,* measures the competing risks for the pathways out of the parental home. Co-residing with one or both parents is the reference category. The pathways are constructed sequentially as follows. We characterise a pathway as *Union* formation if an individual lived in the parental home and was unmarried and not cohabiting in year *t* but if this individual left the home and married, or was cohabiting in year *t* + 1. Leaving home is supposed to be for *Independent* residence if an individual lived in the parental home in year *t* and was not classified into the category Union, but if this individual left the home to reside in a single person household in year *t* + 1. Finally, if an individual lived in the parental home in year *t* and was not classified in the Union category, but if this individual left the parental home to share the residence with other people in year *t* + 1, this pathway is denoted as *Shared* residence.

The variables used in this study are listed and described in Table 1, together with the mean values, SD, minimum and maximum values at the time of the last observation in the person/period setting of the data. Our independent variables include a large set of characteristics of individuals themselves and the parental home. The gender variable is constructed as a dummy variable for woman (1) and men (0). Immigrant origin is specified as dummy variables for six immigrant groups, taking Dutch as the reference category. We also distinguish first- and second-generation migrants by constructing two dummy variables, one for children of two immigrant parents and another for mixed second generation of whom one parent is native Dutch. Individual characteristics include dummy variables for being in education, having graduated in higher education, being employed, and owner-occupied parental home as well as linear variables measuring monthly labour income in 100 euros, and values of dwelling per capita in 1000 euros. Assuming the house price reflects the quality of housing, we use the difference in per capita dwelling prices between origin and destination. This price refers to the value of property as administratively determined on a periodical basis by the municipal authorities in accordance with the Valuation of Immovable Property Act (*WOZ*, in Dutch) which was enacted in 1994. The value of property is estimated for rented and owner-occupied property on the basis of relevant characteristics such as type, size, condition, location, year of construction, as well as information about similar properties recently sold in the neighbourhood. Such an administrative procedure ensures that the estimated administrative price is close to the current market value of the property. An individual-specific dwelling value is defined as the euro value of the dwelling divided by the household size.

Family structure is approximated by two linear variables for the number of children and adults in the household and three marital status dummies taking married as the reference category: one for unmarried, one for widowed and another for divorced. The parental economic position is approximated by dummy variables for labour market attachment of both parents (a dummy for employed, self-employed, welfare benefits and other benefits) and separate linear variables for monthly income of the parents. We also considered local conditions that are measured by the degree of urbanisation of the municipality (dummies for very strongly, strongly and moderate urbanised locations) and two variables for the percentage of non-western migrants and benefit recipients in the neighbourhood.

We present a separate table to show the distribution of variables across the ethnic groups. Table 2 shows mean values of variables by ethnic group at the time of exit or censoring. The mean values of age indicate that a relative large share of Turkish and Moroccan nest leavers leaves the parental home around ages 17 and 18. Many Turkish, Moroccan and Surinamese youth (60–76 %) belong to the second generation, with both parents from the same origin, while most young western second-generation adults have a Dutch parent. Young adults from developing countries differ in a number of characteristics from western migrants and native Dutch people. They are more likely to be enroled in education and less likely to have a job. They live more often in rental homes in ethnically segregated neighbourhoods of highly urbanised municipalities. There is also a concentration of cheap houses and benefit recipients in these neighbourhoods. Young Turkish and Moroccan adults tend to be from the largest households, in which the parents have a relatively weak economic position characterised by low earnings and employment rates and a great dependence on benefits. These households also show indicators of traditional families, such as a high number of children and low rate of labour market participation of the mothers.

**Table 2:** Variables and their ethnic group-specific means at the time of exit or censoring

	Dutch	Moroc	Turk	Surin	Antillian	Oth. non-western	Western
Pathway—union	0.17	0.11	0.11	0.12	0.15	0.11	0.15
Pathway—independent	0.23	0.40	0.42	0.29	0.32	0.31	0.27
Pathway—shared	0.03	0.19	0.16	0.09	0.05	0.08	0.05
Pathway—stayed home	0.57	0.30	0.31	0.50	0.48	0.50	0.53
Age 16	0.01	0.04	0.06	0.03	0.03	0.02	0.02
Age 17	0.05	0.32	0.33	0.14	0.11	0.11	0.07
Age 18	0.09	0.18	0.15	0.11	0.12	0.12	0.10
Age 19	0.11	0.10	0.09	0.09	0.13	0.12	0.12
Age 20	0.10	0.06	0.07	0.08	0.11	0.10	0.10
Age 21	0.64	0.29	0.31	0.55	0.51	0.52	0.60
Girl	0.49	0.50	0.49	0.51	0.49	0.47	0.49
Second generation		0.62	0.76	0.60	0.20	0.26	0.12
Second gen. (mixed)		0.04	0.03	0.16	0.38	0.21	0.68
Individual char							
In education	0.48	0.67	0.63	0.56	0.54	0.63	0.53
Graduated in HE	0.02	0.00	0.00	0.01	0.01	0.01	0.01
Employed	0.52	0.30	0.33	0.35	0.34	0.32	0.42
Monthly income	8.65	4.42	4.93	5.84	6.01	5.75	6.92
Owner-occupied	0.72	0.05	0.19	0.31	0.34	0.29	0.56
Value of dwelling	41.06	19.64	20.19	28.21	32.63	29.00	40.97
Family structure							
No. of adults in HH	1.83	1.85	1.92	1.52	1.55	1.69	1.73
No. of children in HH	1.95	3.08	2.05	1.99	1.95	2.26	1.78
Mother unmarried	0.02	0.00	0.00	0.19	0.22	0.04	0.03
Mother widow	0.02	0.03	0.03	0.02	0.02	0.04	0.03
Mother divorced	0.11	0.09	0.12	0.38	0.25	0.22	0.21
Parents econ.							
Mother employed	0.58	0.11	0.24	0.62	0.59	0.36	0.56
Mother self-employed	0.07	0.00	0.02	0.02	0.03	0.07	0.05
Mother welfare	0.06	0.37	0.39	0.23	0.22	0.31	0.11
Mother benefit	0.04	0.08	0.09	0.06	0.04	0.05	0.06
Father employed	0.72	0.26	0.43	0.46	0.44	0.39	0.59
Father self-employed	0.11	0.02	0.05	0.04	0.04	0.10	0.08
Father welfare	0.06	0.48	0.34	0.13	0.06	0.21	0.08
Father benefit	0.05	0.15	0.09	0.04	0.05	0.05	0.07
Income mother	10.7	4.96	7.83	15.72	14.11	10.12	12.23
Income father	29.59	13.27	15.78	15.36	16.84	15.04	24.69
Local conditions							
Urbanised I	0.09	0.53	0.42	0.58	0.32	0.35	0.18
Urbanised II	0.25	0.25	0.33	0.26	0.40	0.31	0.32
Urbanised III	0.23	0.16	0.17	0.10	0.14	0.18	0.23
Non-western (%)	6.48	32.52	30.95	31.74	20.5	21.56	10.59
μ value houses	1,51,483	99,645	94,030	97,880	1,12,542	1,13,300	2,00,238
Benefits (%)	9.77	15.74	15.79	14.33	12.78	13.08	11.07
*N*	1,32,263	4685	4552	4746	1446	5259	11,362

*Source*: SSD, own calculations

The household structure of Surinamese and Antillean (Caribbean) young adults is clearly different from other non-western migrant groups and sometimes even from Dutch and western migrants. Caribbean youth often lives in households where the mother is most likely unmarried and employed. We observe a significant gender gap in the labour market position between the parents for almost all groups, except Caribbean parents. In general, fathers are more likely to be employed and earn a higher income than mothers. However, a typical Caribbean mother is more likely to work and earn a higher income than the ‘partner’. The gender gap is the strongest for Moroccan and Turkish households. Western migrants have quite similar characteristics to Dutch.

## Pathways Out of the Parental Home

**Fig. 1:**
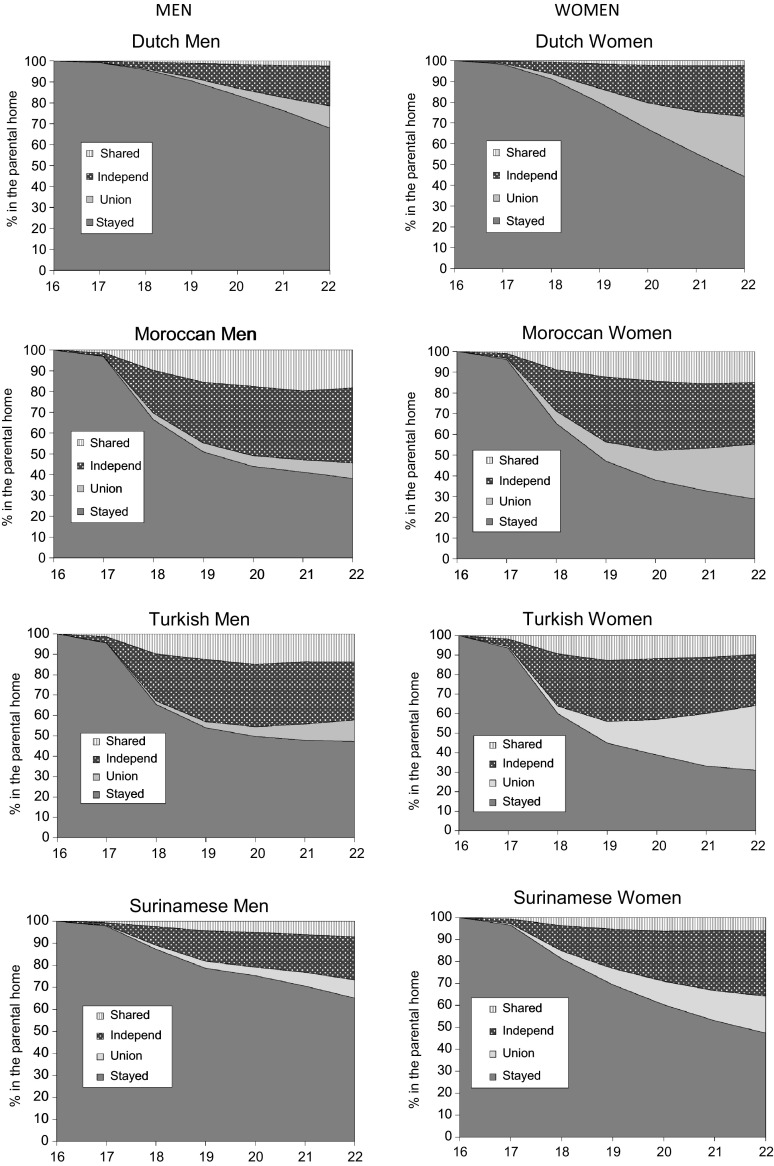
Percentage of young people in the parental home by gender and country of origin

The data provide information on annual changes in individual living arrangements, household characteristics and socio-economic position measured on the last Friday of September each year. Figure 1 shows the ratio between young people living in the parental home and those who leave home for union formation, as well as independent and shared residence by gender for a selected group of origin countries. It is immediately apparent that immigrants leave the parental home earlier than young Dutch people do, and women at younger age than men. Most striking is the substantially earlier departure of Moroccan and Turkish women and men, if they are in the period of transition to formal adulthood age at around 18. Only 26 and 30 % of Moroccan and Turkish women still reside in the parental home when they are 22 years old, compared to 43 % for native Dutch women. A slightly larger proportion of Surinamese women (46 %) still live at home when they are 22. Some 35 and 46 % of all Moroccan and Turkish men live with their parent(s) at age 22. These percentages are again significantly higher at 62 for Surinamese and 67 for Dutch men. In addition to the timing of departure, Turkish and Moroccan men, but particularly women, tend to leave the parental home for union formation more frequently than their Dutch and Surinamese counterparts for whom union formation is of lesser importance.

The ethnic differences in the leaving home pattern shown in Fig. 1 are robust to differences in the socio-economic and socio-demographic characteristics of the youth. We tested this by estimating discrete time duration models with competing risks, using all the variables in Table 1. These models provide a very similar pattern of interethnic differences in the routes and timing of leaving home (the results are not displayed but available on request).

Since this study uses the register data of an entire birth cohort and a competing risks model to estimate the timing and pathways out of home, our results differ from earlier studies on leaving home process of ethnic minorities which used survey data (Bolt 2002; De Valk and Billari 2007; Kleinepier and de Valk 2015). The contradictions between the results of survey and register data can arise from three main sources. First, survey data may be selective, or self-reported retrospective information about the timing of leaving home may be imprecise. Second, register data may not reflect a true process of leaving home because the registered reality may differ from a (unregistered) true situation due to implications of (registered) legal residential address. Third, the contradictions can simply arise from differences in the nature of the analysis and the age composition of the underlying sample.

## Where do Nest Leavers Move to?

The question arises as to whether the choice of destination location is a part of the explanation. Ethnic differences in the characteristics of destination accommodation are estimated by comparing ethnic minority youth to their Dutch counterparts, the reference group. The sample of young Dutch people is the largest in our data, so the estimates of the control variables are possibly largely driven by their characteristics. Table 1b indicates that non-western ethnic minority groups differ from the Dutch youth regarding their parental background and neighbourhood characteristics. We use the degree of urbanisation as a proxy for the geographical mobility of nest leavers. In order to limit the number of destination choices, we constructed a nominal response variable based on the degree of urbanisation. In an attempt to assess the distribution of nest leavers across geographical locations, we defined four typologies of moving direction: moving within the four large cities, moving to more urbanised areas, moving to less urbanised areas and moving within areas with the same degree of urbanisation (but not the four major cities). These typologies are mutually exclusive, and we believe they capture the distance between the parental home and new home, in particular for migrant groups. Since migrants are highly concentrated in strongly urbanised areas, migrants leaving the four large cities and moving to less urbanised areas means long-distance moving.

We estimated the destination of nest leavers using the multinomial logit estimator. Again, we present marginal effects instead of coefficients. Ethnic groups interact with the pathways out of home which makes it possible to identify ethnic differences in the distribution of nest leavers for various pathways across the destinations. The estimated marginal effects for migrant groups by destination show the distribution of nest leavers across four location typologies relative to Dutch independent which is the reference category, holding constant the background characteristics for men and women.

Table 3 (a men, b women) presents the estimated effects of regressors on the distribution of nest leavers across four urbanisation typologies in the form of marginal effects. It is worth mentioning that the pathway-specific ethnic group variables should be compared to the reference group, that is, Dutch nest leavers who move to an independent living arrangement. It is immediately apparent that Turkish and Moroccan nest leavers are more likely to move within the same degree of urbanisation and within the four large cities than Dutch nest leavers. They are relatively less likely to move to more or to less urbanised locations. The distribution of other migrant groups is, in general, quite similar but much less pronounced. An exception is the distribution of Surinamese nest leavers who move to independent or shared accommodation. They are more likely to stay within the four large cities, at the expense of the same urbanisation degree. Migrant groups remain either in the same urbanisation degree or within the four large cities. This pattern is stronger for nest leavers who move to independent or shared accommodation than for nest leavers who opt for union formation. There is no evidence of mobility towards more or less urbanised locations. The difference between first and second generation is small, but mixed second-generation men tend to move to less urbanised locations. When the parents live in owner-occupied homes, the nest leavers often move to more urbanised areas or remain within same urbanisation degree. Nest leavers who study are more likely to move to more urbanised areas.

**Table 3:** Destination of nest leavers 1999–2005, marginal effects: (a) MEN and (b) WOMEN

	Within same urbanisation		Within four cities		To more urbanisation		To less urbanisation	
	MarginE	SE	MarginE	SE	MarginE	SE	MarginE	SE
*(a) MEN*								
Dutch—independent (Ref.)								
Dutch—union	0.06*	0.006	−0.01	0.005	−0.06*	0.006	0.01*	0.004
Dutch—share	0.04*	0.010	0.00	0.007	−0.04*	0.009	0.00	0.006
Moroccan—union	0.14*	0.042	0.05*	0.018	−0.13*	0.050	−0.07*	0.025
Moroccan—independent	0.15*	0.022	0.05*	0.010	−0.13*	0.026	−0.08*	0.013
Moroccan—shared	0.21*	0.029	0.05*	0.012	−0.21*	0.035	−0.05*	0.015
Turkish—union	0.08	0.045	0.03	0.019	−0.08	0.054	−0.03	0.020
Turkish—independent	0.24*	0.023	0.03*	0.010	−0.22*	0.027	−0.05*	0.012
Turkish—shared	0.33*	0.036	0.03*	0.014	−0.32*	0.046	−0.05*	0.017
Surinamese—union	0.02	0.038	0.03	0.016	−0.01	0.041	−0.04*	0.018
Surinamese—independent	0.00	0.025	0.04*	0.011	−0.04	0.026	0.00	0.011
Surinamese—shared	0.04	0.045	0.06*	0.016	−0.11*	0.049	0.01	0.016
Antillean—union	−0.02	0.048	−0.03	0.026	0.05	0.050	0.01	0.019
Antillean—independent	0.04	0.033	0.04*	0.016	−0.04	0.034	−0.04*	0.019
Antillean—shared	0.14*	0.070	0.03	0.031	−0.14	0.077	−0.03	0.037
Other non-west—union	0.03	0.030	0.03*	0.015	−0.05	0.031	−0.01	0.015
Other non-west—independent	0.01	0.017	0.02	0.009	0.01	0.017	−0.03*	0.009
Other non-west—shared	0.04	0.029	0.02	0.015	−0.01	0.031	−0.05*	0.017
Western—union	0.06*	0.022	0.00	0.013	−0.04	0.021	−0.02	0.012
Western—independent	0.01	0.017	0.02*	0.009	−0.01	0.017	−0.02*	0.009
Western—shared	0.11*	0.028	0.01	0.016	−0.08*	0.028	−0.05*	0.018
Second generation	−0.01	0.015	0.01	0.006	0.00	0.016	0.00	0.008
Second generation (mixed)	−0.02	0.016	0.01	0.008	−0.01	0.016	0.03*	0.009
No. of children in household	−0.01	0.006	−0.01	0.004	0.01	0.007	0.01*	0.004
No. of adults in household	0.02*	0.006	−0.01*	0.003	0.00	0.006	−0.01*	0.003
Owner	0.04*	0.010	−0.13*	0.006	0.11*	0.009	−0.02*	0.005
In education	−0.16*	0.007	0.01*	0.004	0.16*	0.007	−0.02*	0.004
Income	0.00*	0.001	0.00*	0.000	0.00*	0.001	0.00*	0.000
Housing value	0.00*	0.000	0.00*	0.000	0.00*	0.000	0.00*	0.000
Mother income	0.00*	0.000	0.00*	0.000	0.00	0.000	0.00	0.000
Father income	0.00*	0.000	0.00*	0.000	0.00*	0.000	0.00	0.000
Employed	0.04*	0.008	0.02*	0.005	−0.06*	0.008	0.00	0.005
Mother unmarried	0.00	0.015	0.06*	0.008	−0.06*	0.015	0.00	0.008
Mother widow	0.05*	0.022	−0.03*	0.014	−0.01	0.021	−0.01	0.012
Mother divorced	0.04*	0.008	0.01	0.005	−0.04*	0.008	0.00	0.004
Mother employed	0.01	0.025	0.01	0.017	0.00	0.022	−0.02	0.015
Mother self-employed	0.00	0.024	0.01	0.016	0.00	0.021	−0.01	0.015
Mother in welfare	0.04	0.026	0.02	0.017	−0.04	0.023	−0.01	0.016
Mother in benefits	0.02	0.027	0.02	0.018	−0.01	0.024	−0.02	0.016
Father employed	−0.05*	0.018	0.05*	0.011	0.03	0.016	−0.03*	0.011
Father self-employed	−0.06	0.019	0.05	0.011	0.04	0.016	−0.03	0.012
Father in welfare	−0.05	0.020	0.07	0.012	0.02	0.018	−0.04	0.012
Father in benefits	−0.07	0.022	0.05	0.013	0.04	0.020	−0.02	0.013
Non-western in neigh (%)	0.00	0.000	0.01	0.000	−0.01	0.000	0.00	0.000
Mean house value in neigh	0.00	0.000	0.00	0.000	0.00	0.000	0.00	0.000
Benefits in neigh (%)	0.01	0.001	0.00	0.001	−0.01	0.001	0.00	0.000
*(b) WOMEN*								
Dutch—union	0.14*	0.005	−0.02*	0.003	−0.15*	0.004	0.03*	0.003
Dutch—share	0.02	0.010	0.00	0.006	−0.02*	0.009	0.00	0.006
Moroccan—union	−0.03	0.032	0.03*	0.011	0.02	0.034	−0.02	0.014
Moroccan—independent	0.13*	0.022	0.03*	0.008	−0.08*	0.025	−0.09*	0.013
Moroccan—shared	0.17*	0.031	0.04*	0.011	−0.12*	0.035	−0.09*	0.019
Turkish—union	0.09*	0.030	−0.01	0.012	−0.02	0.034	−0.05*	0.016
Turkish—independent	0.21*	0.022	0.01	0.008	−0.14*	0.024	−0.08*	0.013
Turkish—shared	0.34*	0.041	0.03*	0.013	−0.30*	0.051	−0.07*	0.021
Surinamese—union	0.01	0.030	0.00	0.011	0.02	0.031	−0.02	0.014
Surinamese—independent	−0.05*	0.023	0.02*	0.008	0.04	0.022	−0.01	0.010
Surinamese—shared	−0.02	0.041	0.02	0.013	−0.01	0.041	0.01	0.016
Antillean—union	0.13*	0.040	−0.01	0.018	−0.14*	0.043	0.02	0.018
Antillean—independent	0.07*	0.029	−0.03	0.013	−0.03	0.029	−0.01	0.015
Antillean—shared	0.42	17.456	0.16	6.366	0.39	11.450	−0.97	35.271
Other non-west—union	0.03	0.024	0.01	0.011	−0.04	0.024	−0.01	0.012
Other non-west—independent	0.01	0.018	0.00	0.008	0.03	0.017	−0.03*	0.010
Other non-west—shared	0.04	0.032	0.00	0.013	0.00	0.032	−0.04*	0.018
Western—union	0.12*	0.016	−0.01	0.009	−0.10*	0.016	−0.01	0.010
Western—independent	−0.02	0.016	0.01*	0.008	0.01	0.015	−0.01	0.009
Western—shared	0.03	0.029	0.00	0.014	−0.01	0.027	−0.02	0.017
Second generation	0.00	0.014	0.02*	0.005	−0.02	0.015	0.01	0.007
Second generation (mixed)	0.00	0.014	0.01	0.006	−0.02	0.013	0.01	0.008
No. of children in household	−0.01	0.006	−0.01*	0.003	0.01*	0.006	0.00	0.003
No. of adults in household	0.02*	0.005	−0.01*	0.003	−0.01*	0.005	0.00	0.003
Owner	0.07*	0.008	−0.11*	0.005	0.09*	0.008	−0.04*	0.005
In education	−0.12*	0.006	0.01*	0.003	0.13*	0.006	−0.02*	0.003
Income	0.00*	0.001	0.00	0.000	0.00*	0.001	0.00	0.000
Housing value	0.00*	0.000	0.00*	0.000	0.00*	0.000	0.00*	0.000
Mother income	0.00*	0.000	0.00*	0.000	0.00*	0.000	0.00	0.000
Father income	0.00*	0.000	0.00*	0.000	0.00*	0.000	0.00*	0.000
Employed	0.04*	0.007	0.01*	0.004	−0.05*	0.007	0.00	0.004
Mother unmarried	0.01	0.014	0.04*	0.006	−0.06*	0.014	0.01	0.007
Mother widow	0.07*	0.019	−0.02	0.010	−0.05*	0.018	0.01	0.011
Mother divorced	0.03*	0.007	0.00	0.004	−0.04*	0.007	0.00	0.004
Mother employed	−0.03	0.021	0.03	0.013	0.00	0.019	0.00	0.014
Mother self-employed	−0.02	0.021	0.02	0.012	0.01	0.018	−0.01	0.013
Mother in welfare	0.01	0.022	0.05*	0.013	−0.06*	0.020	−0.01	0.014
Mother in benefits	−0.03	0.024	0.03*	0.014	0.01	0.021	−0.01	0.015
Father employed	−0.08*	0.017	0.04*	0.009	0.07*	0.014	−0.02	0.011
Father self-employed	−0.09*	0.017	0.05*	0.009	0.06*	0.014	−0.02*	0.011
Father in welfare	−0.06*	0.018	0.04*	0.010	0.04*	0.016	−0.02*	0.011
Father in benefits	−0.10*	0.020	0.05*	0.011	0.06*	0.018	−0.02	0.012
Non-western in neigh (%)	0.00*	0.000	0.01*	0.000	−0.02*	0.000	0.00*	0.000
Mean house value in neigh	0.00*	0.000	0.00*	0.000	0.00*	0.000	0.00*	0.000
Benefits in neigh (%)	0.01*	0.001	0.00	0.000	−0.01*	0.001	0.00*	0.000

*Source*: SSD, own calculations

* Significant at 5 %

To show potential ethnic differences in destination choice, we firstly calculate the probabilities of moving to four directions for Dutch and migrant groups, relying on the mean values of the observed characteristics. Differences in these probabilities represent the gap between Dutch and migrant groups, given their observed characteristics. Subsequently, we calculate the predicted probabilities for each migrant group per outcome attaching the characteristics of Dutch to each migrant group. This gives a prediction of the ethnic component: the difference between the probability of moving to a destination for Dutch (D) minus the probability of moving for a migrant group (for instance for Moroccans) if Moroccans had taken the characteristics of Dutch (i.e. *M*(*D*)). Table 4 presents the probabilities of moving in four directions and the ethnic gap in the probabilities by migrant group. The predictions indicate that Turkish, Moroccan and Surinamese nest leavers are most likely to move within four large cities and are less likely to move to more urbanised locations, compared to Dutch nest leavers. About half of migrant nest leavers move within the four large cities versus 8 % of Dutch nest leavers. By contrast, only 6, 8 and 14 % of Turkish, Moroccan and Surinamese men move to more urbanised locations, compared to 48 % of Dutch men. If these migrant groups had Dutch characteristics, their probability would be around 15 % whereby the ethnic gap is 32 and 34 %. For women, the distribution of movers is quite similar.

**Table 4:** Ethnic gap in the conditional probabilities of moving across various urbanisation degrees

	MEN				WOMEN			
	Same urb.	Four cities	More urb.	Less urb.	Same urb.	Four cities	More urb.	Less urb.
Dutch (*D*)	0.37	0.08	0.48	0.07	0.41	0.07	0.44	0.08
Moroccan (*M*)	0.37	0.51	0.08	0.04	0.31	0.53	0.10	0.05
Moroccan as Dutch (*M*(*D*))	0.29	0.42	0.14	0.14	0.28	0.44	0.11	0.16
Ethnic gap (*D*–*M*(*D*))	0.07	−0.34	0.34	−0.07	0.13	−0.36	0.32	−0.09
Turkish (*T*)	0.47	0.42	0.06	0.05	0.44	0.42	0.09	0.05
Turkish as Dutch (*T*(*D*))	0.32	0.39	0.16	0.13	0.32	0.39	0.15	0.15
Ethnic gap (*D*–*T*(*D*)	0.05	−0.31	0.32	−0.07	0.09	−0.31	0.29	−0.07
Surinamese (*S*)	0.23	0.53	0.14	0.09	0.20	0.51	0.19	0.10
Surinamese as Dutch (*S*(*D*))	0.26	0.45	0.16	0.13	0.25	0.46	0.16	0.14
Ethnic gap (*D*–*S*(*D*))	0.11	−0.37	0.32	−0.06	0.17	−0.39	0.28	−0.06

*Source*: SSD, own calculations

The probabilities of moving within the same urbanisation degree is 37 % for Dutch and Moroccan men, higher for Turkish men (47 %) but lower for Surinamese men (23 %). If the migrant groups had taken on Dutch characteristics, their probability of moving within the same urbanisation would be around 30 %, whereby the ethnic gap is 5, 7, and 11 % for Turkish, Moroccan and Surinamese male nest leavers. The probability of moving to less urbanised locations is, in general, small and that is to be expected for the youth being studied. This probability is 7 % for Dutch and 4, 5 and 9 % for Moroccan, Turkish and Surinamese men. If these migrant groups had Dutch characteristics, their probability would be about 14 %. This suggests that the probability of moving to less urbanised locations would be about 7 % higher for migrant groups compared to Dutch if they had the Dutch characteristics.

## Quality of Destination Housing

The price of a dwelling for an individual captures the most relevant characteristics of the dwelling and thereby is a good proxy for the quality of the home. Table 5 displays the mean individual-specific value of the parental home and destination home in thousands of euros by gender, ethnicity and the pathway out of a home. It is immediately apparent that the value of home for Dutch youth in the parental home is twice as high as that for Turkish and Moroccan youth, i.e. about 20 versus 40 thousand euros. The individual value of the parental home for Surinamese and other non-western youth is about 28 thousands of euros. The home value of western youth is pretty similar to that of the Dutch youth. Nest leavers from Dutch and western households move to a destination home with a lower value, while ethnic minority nest leavers, in particular Turkish and Moroccan nest leavers, improve their home quality. After some convergence, the home value of Dutch and western nest leavers in the destination is still substantially higher compared to non-western migrants, in particular Turkish and Moroccan nest leavers. The pattern of ethnic differences in the change in home values is quite similar for men and women.

**Table 5:** Mean individual-specific values of dwellings in the origins and destinations of nest leavers in 1000 euros

	MEN			WOMEN		
	Union	Indep.	Shared	Union	Indep.	Shared
Dutch						
Origin	41.0	48.5	47.4	38.4	46.4	47.1
Destination	38.8	43.0	37.1	42.1	40.7	36.0
Moroccan						
Origin	20.0	19.0	21.9	19.6	19.4	20.9
Destination	27.6	21.5	25.7	27.7	22.5	24.5
Turkish						
Origin	20.5	20.9	22.1	18.6	20.0	21.8
Destination	27.8	23.0	22.9	28.5	22.0	22.7
Surinamese						
Origin	27.0	28.8	29.7	27.8	28.7	27.3
Destination	29.2	31.7	30.3	31.6	31.7	27.4
Antillean						
Origin	29.2	38.6	37.3	31.6	30.4	30.8
Destination	31.3	41.0	26.2	34.6	32.0	30.7
Other non-west						
Origin	27.5	29.3	28.6	31.8	29.8	26.0
Destination	30.8	34.8	30.6	34.8	32.0	28.2
Western						
Origin	40.1	50.1	45.4	38.8	46.3	43.4
Destination	37.1	41.9	38.0	38.4	40.8	32.8

*Source*: SSD, own calculations

In addition, we conducted a regression analysis to explain ethnic differences in the change in housing quality. A positive value for the change in individual-specific price of dwelling after leaving home, a negative value denotes a decrease in home quality for nest leavers. To deal with potential selection problems, we estimate a Heckman selection model which includes a selection equation in addition to the outcome equation. In addition to the common variables, the selection equation includes dummies for the labour market status of both parents (employed, self-employed, welfare and benefit) and the country of origin fixed effects and the age of leaving home dummies, while the outcome equation additionally uses the interactions between the country of origin and the pathways out of the parental home. Table 6 shows the estimated parameters for men and women from two-step Heckman selection models. In addition to these variables, the selection equations which are not presented here include dummies for the labour market status of both parents (employed, self-employed, welfare and benefit) and the country of origin fixed effects and the age of leaving home dummies.

**Table 6:** Parameter estimates of the change in per capita value of the home after leaving home (in 1000 s)

	MEN				WOMEN			
	OLS		Selection		OLS		Selection	
Dutch—independent (Refer.)								
Dutch—union	4.663***	0.816	4.677***	0.797	−0.959*	0.431	−0.829*	0.423
Dutch—share	5.183***	1.195	5.808***	1.166	4.883***	0.829	4.815***	0.811
Moroccan—union	3.977	3.932	−27.273***	4.204	6.335**	2.015	−7.722***	2.145
Moroccan—independent	9.239***	2.123	−22.748***	2.582	9.180***	1.450	−4.803**	1.628
Moroccan—shared	5.994*	2.546	−25.003***	2.927	8.009***	1.861	−6.396**	1.999
Turkish—union	4.106	4.138	−24.995***	4.376	2.574	2.078	−11.376***	2.211
Turkish—independent	3.615	2.157	−22.893***	2.550	4.917***	1.459	−8.197***	1.640
Turkish—shared	6.287*	2.787	−20.799***	3.097	5.495**	2.095	−7.952***	2.210
Surinamese—union	3.518	3.764	−4.997	3.866	0.001	2.072	−3.856	2.155
Surinamese—independent	−1.772	2.546	−8.792**	2.741	−2.333	1.559	−5.431**	1.684
Surinamese—shared	−0.424	3.873	−6.585	3.956	1.044	2.542	−2.506	2.585
Antillean—union	4.684	5.427	−3.695	5.578	−1.800	3.262	−5.241	3.369
Antillean—independent	−2.102	3.911	−11.265**	4.184	−0.637	2.359	−4.646	2.542
Antillean—shared	9.760	7.824	0.129	7.921	0.052	6.250	−3.496	6.240
Other non-west—union	1.561	3.436	−12.772***	3.541	1.595	1.896	−2.119	1.961
Other non-west—independent	−1.304	1.993	−14.936***	2.203	0.342	1.457	−3.753*	1.559
Other non-west—shared	3.032	3.379	−10.820**	3.474	1.949	2.513	−1.314	2.534
Western—union	3.582	2.674	−2.694	2.782	0.691	1.395	−1.426	1.490
Western—independent	1.198	2.039	−5.809**	2.212	−1.130	1.312	−3.575*	1.415
Western—shared	3.634	3.389	−2.569	3.449	5.142*	2.347	2.480	2.371
Second generation	−0.452	1.429	2.849	1.618	−0.313	0.947	1.195	1.072
Second generation (mixed)	0.597	1.853	5.728**	2.056	0.833	1.135	2.256	1.270
No. of adults in household	−1.700**	0.654	5.682***	0.774	−0.750	0.398	4.278***	0.465
No. of children in household	−4.334***	0.237	−4.762***	0.264	−4.554***	0.142	−7.006***	0.172
Owner	5.739***	0.796	1.980*	0.875	5.890***	0.443	2.049***	0.502
In education	8.683***	0.895	7.454***	0.980	6.270***	0.533	9.270***	0.602
Log income	−2.914***	0.420	−6.286***	0.450	−3.402***	0.245	−5.209***	0.263
Log income mother	0.447	0.491	−1.904***	0.535	1.692***	0.288	0.148	0.316
Log income father	3.357***	0.577	−0.335	0.640	3.581***	0.350	2.137***	0.385
Employed	−2.637**	1.023	2.241*	1.124	−3.230***	0.597	−4.279***	0.662
Mother unmarried	−2.118	1.796	−4.211*	2.000	−0.887	1.097	−2.628*	1.234
Mother widow	3.011	2.352	13.628***	2.631	2.647*	1.344	6.974***	1.515
Mother divorced	1.467	0.979	−1.116	1.094	2.630***	0.592	0.304	0.666
Urbanised most strongly	2.865*	1.158	7.267***	1.289	3.055***	0.693	9.395***	0.792
Urbanised strongly	0.472	0.818	1.234	0.906	0.286	0.471	3.536***	0.538
Urbanised moderate	1.017	0.846	0.562	0.934	0.042	0.481	1.234*	0.542
Non-western (%)	0.134***	0.033	0.116**	0.037	0.075***	0.021	0.054*	0.023
Ln (value of houses in neigh)	20.063***	0.769	16.335***	0.774	18.599***	0.469	16.106***	0.472
Benefits in neigh (%)	−0.058	0.096	−0.366***	0.105	0.042	0.058	−0.057	0.064
Constant	−90.953***	5.639	74.794***	8.072	−85.421***	3.364	16.857***	4.466
Mills lambda			−57.350***	2.009			−41.876***	1.100
*R* ^2^	0.090				0.139			
*N*	24,048		5,07,830		38,433		4,83,676	
*N*-censored			4,83,782				4,45,243	

*Source*: SSD, own calculations

* *p* < 0.05; ** *p* < 0.01; *** *p* < 0.001

The selection term lambda is significant and negative which suggests that (unobserved) factors that make leaving home more likely are associated with lower differences in the values of origin and destination housing. In other words, young people are more likely to leave the parental home if the gap in the quality of the home in the origin and destination is lower.

The ordinary least squares estimates for men and women indicate that, in general, Moroccan and Turkish nest leavers face a positive difference (5–10 thousand euros) in the change in the price of the home with respect to Dutch nest leavers who move to an independent living arrangement. This positive difference indicates a higher decrease in the quality of the home for Moroccan and Turkish nest leavers compared to Dutch nest leavers. The positive differences are, however, not statistically significant for Turkish men leaving home for an independent living arrangement, Moroccan men for union formation, and Turkish men and women leaving home for union formation. Differences in the change in home value are not significant for other ethnic minority groups and Dutch nest leavers who opt for an independent living arrangement.

Ethnic differences in the change in home quality after leaving home turned out to be the opposite for some important variables once the estimates are corrected for selectivity in leaving home process. The selection models generated much higher negative differences for Turkish and Moroccan nest leavers with respect to Dutch nest leavers who move to an independent living arrangement. Larger negative differences have also been found for other ethnic minority groups such as other non-western, Surinamese, Antillean and western nest leavers, although the estimates for these groups are not always statistically significant. Ethnic differences in the change in home quality after leaving home are drastically lower for ethnic minority women compared to ethnic minority men. The selection effect is obviously important, in particular for male nest leavers. The leaving home decision of women is relatively weakly correlated with a change in the home quality. The estimated negative differences for the ethnic minority groups, in particular Turkish and Moroccan nest leavers, suggest a relative improvement in the home quality after leaving home for these groups, relative to Dutch nest leavers. This is in fact a confirmation of the second hypothesis.

Second-generation minorities move to a lower quality home compared to their first-generation counterparts. Nest leavers from a densely child-populated household are better off if they leave the parental home, while those from a household with more adults are worse off. Nest leavers from an owner-occupied parental home are worse off in their new home. As expected, leaving home for study is strongly associated with low-quality housing. A better socio-economic position of the nest leaver leads to better destination housing. The effect of parental income is differentiated for men and women: a higher income level of the mother is associated with a decline in housing quality for daughters, while a higher income of the father is associated with a more pronounced decline in housing quality of sons and daughters. The position of mother in the household is also an important factor. The children of unmarried mothers leave the parental home for a better home, while the children of widowed mothers experience a drastic decline in the quality of home, compared to children of married mothers, the reference group. Nest leavers in the most strongly urbanised locations (read the four big cities) are substantially worse off. In addition to individual characteristics, the quality of neighbourhood appears to be an important predictor of the quality of destination home. The mean monetary value of houses in the neighbourhood is a dominant indicator of neighbourhood quality. Nest leavers from better neighbourhoods experience a drastic decline in housing quality. The predictive power of neighbourhood ethnic composition and welfare dependency is estimated to be limited.

## Conclusions

This paper investigated the role of the destination accommodation in addition to individual characteristics and parental background in explaining interethnic differences in leaving home. The impact of the destination accommodation is approximated in two ways. *Firstly*, we considered the location choice of nest leavers across various degrees of urbanisation which largely reflect the distance from the parental home. Hypothesis 1 suggests that ethnic minority young people will move further away from the parental home and at a younger age to evade parental control. In Dutch society in which they grow up, they may experience more intensive conflicts with their parents who may adhere to home country standards that are usually more authoritarian and family-oriented. *Second*, we have investigated the quality of the destination accommodation relative to the parental home as reflected by the per capita price of the accommodation. The difference in prices between the destination and parental home may reflect a relative advantage of leaving home. Hypothesis 2 indicates that young ethnic minority adults leave home at younger ages than their native Dutch counterparts because they can improve their living conditions significantly after leaving home for an ‘independent’ living arrangement. Non-western households on average include a higher number of household members, and they usually live in small rental homes in less appealing neighbourhoods. The quality of their parental home is then relatively low, so home conditions after leaving home may be better.

Using longitudinal register data, this study has shown that ethnic minority groups, in particular young Turkish and Moroccan adults, leave the parental home earlier than their Dutch counterparts to form a union or to live independently or with others. Strikingly, immigrant women and men often leave home to live independently and much less frequently for union purposes. The analysis of the location of the new destination indicates that interethnic differences in home-leaving behaviour are only weakly related to the level of urbanisation of the destination housing. Turkish and Moroccan youth leave home at younger ages and for accommodation close to the parental home. More precisely, they are more likely to remain in the four large cities and in places with the same degree of urbanisation as the location of the parental home. Consequently, for nest leavers from traditional families, leaving home cannot necessarily be associated with an attempt to evade parental authority. This result leads to a rejection of the first hypothesis.

Our analysis of differences in the quality of the destination and the parental home indicates that non-western groups, in particular young Turkish and Moroccan adults, are substantially better off after leaving home than Dutch nest leavers. The analysis provides strong evidence of the selectivity of nest leavers. Young people are more likely to leave the parental home if they do not suffer from great home quality loss or if they are going to be better off. Our best selectivity-corrected estimates suggest that Turkish and Moroccan male nest leavers show significantly more improvement in their dwelling value after leaving their parental home than Dutch nest leavers. This convergence in the quality gap is composed by both an improvement of dwelling value among Turkish and Moroccan male nest leavers and a decrease in value for Dutch male nest leavers. These results confirm the second hypothesis: Nest leavers from ethnic minority groups improve their housing quality by leaving home at younger ages compared to their Dutch counterparts. Our findings suggest that the relative improvement in the home quality for Turkish and Moroccan nest leavers may explain this counterintuitive phenomenon.

Our results using register data show contradictions in the leaving home patterns of ethnic minorities based on survey research (Bolt 2002; De Valk and Billari 2007). Both studies rely on the household samples with limited geographical coverage, a broad age range and non-response. However, register data can also be biased. In the Netherlands, students who do not live with parent(s) are eligible for a higher amount of scholarship than co-residing students. This can be an incentive for using another address while living with parents. We do not know whether students from various ethnic origins respond differently to this arrangement. Furthermore, a large segment of the non-western ethnic minority groups live in social housing in large cities where the quality of housing is relatively low (Zorlu et al. 2014). It might be expected that youth from disadvantaged ethnic minority groups are offered a rental home in the social housing sector to improve their housing quality more often and earlier in life than Dutch young adults. At the same time, the young family members of these tenants are in favourable position to be eligible for a new house in this segment, because of their long residence is counted as waiting time. An apartment in the public housing segment can be used by another family member or even illegally sub-leased for a much higher rent while co-residing with parents. Our estimates could be biased if such informal practices occur disproportionally across ethnic groups. But there is no empirical evidence to prove these assumptions.

In sum, this study contributes to the literature by an analytical integration of the choice of the destination home into the leaving home decisions and providing evidence on the role of the quality and location of the destination home in the leaving home process of the ethnic minority groups in the Netherlands.

## References

[CR1] Aassve A, Billari FC, Mazzuco S, Ongaro F (2002). Leaving home: A comparative analysis of ECHP data. Journal of European Social Policy.

[CR2] Aquilino WS (1991). Family structure and home-leaving: A further specification of the relationship. Journal of Marriage and the Family.

[CR3] Bakker BFM, Van Rooijen J, Van Toor L (2014). The system of social statistical datasets of Statistics Netherlands: An integral approach to the production of register-based social statistics. Statistical Journal of the IAOS.

[CR4] Bernhardt E, Gahler M, Goldscheider F (2005). Childhood family structure and routes out of the parental home in Sweden. Acta Sociologica.

[CR5] Billari FC, Liefbroer AC (2007). Should I stay or should I go? The impact of age norms on leaving home. Demography.

[CR6] Bolt G (2002). Turkish and Moroccan couples and their first steps on the Dutch housing market: Co-residence or independence?. Journal of Housing and the Built Environment.

[CR7] Buck N, Scott J (1993). She’s leaving home: But why? An analysis of young people leaving the parental home. Journal of Marriage and the Family.

[CR34] Bucx F, van Wel F, Knijn T, Hagendoorn L (2008). Intergenerational contact and the life course status of young adult children. Journal of Marriage and Family.

[CR8] De Beer, J. (2003). *Immigratie uit Europese Unie hangt samen met conjunctuur*. CBS. http://www.cbs.nl/NR/rdonlyres/1C5B8762-5927-453E−8158-8CF6B5153ADB/0/2004k2b15p043art.pdf.

[CR10] De Valk HAG, Billari FC (2007). Living arrangements of migrant and Dutch young adults: The family influence disentangled. Population Studies.

[CR11] De Valk HAG, Liefbroer AC (2007). Timing preferences for women’s family-life transitions: Intergenerational transmission among migrants and Dutch. Journal of Marriage and Family.

[CR12] De Valk HAG, Liefbroer AC (2007). Parental influence on union formation preferences among Turkish, Moroccan, and Dutch Adolescents in the Netherlands. Journal of Cross-Cultural Psychology.

[CR13] Ermisch J (1999). Prices, parents and young people’s household formation. Journal of Urban Economics.

[CR14] Giuliano P (2007). Living arrangements in western Europe: Does cultural origin matter?. Journal of the European Economic Association.

[CR15] Glick JE, Van Hook J (2002). Parents’ coresidence with adult children: Can immigration explain racial and ethnic variation?. Journal of Marriage and Family.

[CR16] Goldscheider F, Thornton A, Young-DeMarco L (1993). A portrait of the nest leaving process in early adulthood. Demography.

[CR17] Kleinepier T, De Valk HAG (2015). Wie speelt een rol in het verlaten van het ouderlijk huis van Marokkanen en Turken?. Demos: Bulletin over Bevolking en Samenleving.

[CR18] Knijn TCM, Liefbroer AC, Dykstra PA, Kalmijn M, Knijn T, Komter A, Liefbroer A, Mulder CH (2006). More than kind: Instrumental support in families. Family solidarity in the Netherlands.

[CR19] Koc I (2007). The timing of leaving parental home and its linkages to other life course events in Turkey. Marriage and Family Review.

[CR33] Leopold T, Geißler F, Pink S (2012). How far do children move? Spatial distances after leaving the parental home. Social Science Research.

[CR20] Mulder CH (2013). Family dynamics and housing: Conceptual issues and empirical findings. Demographic Research.

[CR21] Mulder CH, Clark WAV (2000). Leaving home and leaving the state: Evidence from the United States. International Journal of Population Geography.

[CR22] Mulder CH, Hooimeijer P (2002). Leaving home in the Netherlands: Timing and first housing. Journal of Housing and the Built Environment.

[CR23] Mulder CH, van der Meer MJ (2009). Geographical distances and support from family members. Population, Space and Place.

[CR24] Murphy M, Wang D (1998). Demographic and socio-economic influences on patterns of leaving home in post-war Britain. Demography.

[CR25] Nilsson K, Strandh M (1999). Nest leaving in Sweden: The importance of early educational and labor market careers. Journal of Marriage and the Family.

[CR26] Schans, D. (2007). *Ethnic diversity in intergenerational solidarity*. Dissertation thesis. Interuniversity Center for Social Science Theory and Methodology, Universiteit Utrecht.

[CR27] Whittington LA, Peters HE (1996). Economic incentives for financial and residential independence. Demography.

[CR28] Zorlu A (2013). Occupational adjustment of immigrants in the Netherlands. Journal of International Migration and Integration.

[CR29] Zorlu A, Mulder CH (2008). Initial and subsequent location choices of immigrants to the Netherlands. Regional Studies.

[CR30] Zorlu A, Mulder CH (2010). Location choices of migrant nest leavers: Spatial assimilation or continued segregation?. Advances in Life Course Research.

[CR31] Zorlu A, Mulder CH (2011). Ethnic differences in leaving home: Timing and pathways. Demography.

[CR32] Zorlu A, Mulder CH, Van Gaalen R (2014). Ethnic disparities in the transition to home ownership. Journal of Housing Economics.

